# Are patient ratings of interactions with providers and health plans associated with technical quality of care in systemic lupus erythematosus?

**DOI:** 10.1186/ar4619

**Published:** 2014-09-18

**Authors:** Edward Yelin, Laura Trupin, Jinoos Yazdany

**Affiliations:** 1 University of California, San Francisco, San Francisco, CA, USA

## Background

Prior research has shown that the technical quality of SLE care is associated with the degree of subsequent accumulated damage. However, it is not known whether the nature of interactions between patients and providers and health systems is associated with the technical quality of care.

## Methods

We analyzed data from the UCSF Lupus Outcomes Study (LOS), a national sample of persons with SLE interviewed annually using a structured telephone survey. The survey includes batteries from the Consumer Assessment of Health Plans developed by the US Agency for Healthcare Research and Quality and the Interpersonal Processes of Care Scales to rate care along six dimensions about providers (patient-provider communication, shared decision-making, and trust) and health systems (promptness/timeliness of care, care coordination, and assessment of health plans) from 0 to 100. Due to the fact that the ratings were not normally distributed, we dichotomized the measures at the lowest versus the highest three quartiles. The survey also includes the 13 quality indicators (QIs) for SLE that can be reliably reported by patients. The QIs were aggregated into a pass rate, defined as the number of QIs received as a proportion of those for which individuals are eligible. We used generalized estimating equations to model the relationship of the QI pass rate with being in the lowest quartile of ratings of each individual dimension and with being in the lowest quartile on zero, one to three, and four to six of the dimensions. Models were adjusted for age, race/ethnicity, education, poverty status, presence and kind of health insurance, specialty of principal SLE physician, disease duration, disease activity (SLAQ), and disease damage (BILD).

## Results

A total of 640 LOS participants with ≥1 visit to their principal SLE provider in the year prior to interview were eligible for analysis. Mean age was 52.8 ± 12.6 years and mean disease duration was 20.1 ± 8.8 years; 38% were nonwhites, and 14% were in poverty. Being in the lowest quartile of ratings on any one individual dimension was not associated with a statistically significant difference in QI pass rates (data not shown). Being in the lowest quartile of ratings on four to six dimensions was associated with significantly lower pass rates (0.63 vs. 0.71 for those in the lowest quartile on no dimensions, *P *= 0.02) (Table 1).

**Table 1:** Technical quality of care pass rates by ratings of healthcare experiences in SLE, aggregate

Number of dimensions	QI pass rate (95% CI)
None	0.71 (0.68, 0.74)
One to three	0.70 (0.67, 0.72)
Four to six	0.63 (0.58, 0.68)

## Conclusions

Low ratings on multiple dimensions of interactions may be a sentinel for poor technical quality of care. In the USA, ratings of providers and health plans are in the public domain and this information can help persons with SLE choose providers and health plans more likely to achieve high technical quality of care.

